# Proceedings of the Lippe Society

**Published:** 1889-05

**Authors:** Geo. H. Clark

**Affiliations:** Secretary


### PROCEEDINGS OF THE LIPPE SOCIETY.

The 130th meeting of the Lippe Society was held on Tuesday evening, March 12th. Dr. C. Carleton Smith occupied the chair. The minutes of the last meeting were read and approved.

Dr. Farley then reported that the case published at p. 75, HOMŒOPATHIC PHYSICIAN for February was given one dose of Phos.$^{cm}$, after which there was one paroxysm of pain, and none since.

Dr. James—With the permission of the members, I should like to turn aside from the regular order of business, and offer a few remarks upon the question, Is the homœopathic remedy sufficient in relieving suffering? Allopaths and mongrels always maintain that we are unable to relieve pain without Morphia. They say Morphia is the great stand-by; is God's great gift, etc. In February, 1881, Dr. Lippe was extremely ill, and continued sick for over two months. I was in the charge of his practice at the time. A few weeks previously a telegram had come to Dr. Lippe, from a physician in Detroit, asking for a remedy for a case of renal colic. The patient was a lady who had been under old-school treatment. She had then fallen into the hands of the homœopathist from whom the telegram came. She had been given Morphia and Ether by both the old-school doctor and a mongrel homœopath, without benefit. She had passed numbers of large calculi, and Dr. Lippe advised Lycopodium. She got almost instant relief. The lady thought it better to be where the physician resided who could give her relief so readily, so she came to Philadelphia. Owing to Dr. Lippe's illness, I took charge of her. The next attack came on one evening. I found her writhing. Her sister was on the bed pressing the right side of the patient, who was constantly crying for harder pressure. She was groaning horribly; although the pain was aggravated by slight touching, hard pressure would relieve. I gave her Nux vomica$^{200}$, which made her quiet in a few moments. She then asked, "What makes me so quiet? the pain is no better. Did you give me Morphia?" "No, I am a homœopathician. I have given you a high potency of a homœopathic remedy." She said, "If you have given me Morphia I shall know it, and I shall immediately send you away, for it has such a bad effect that I shall never take it again." The relief from the Nux vomica was striking and singularly prompt. Eventually she had abscesses in the kidneys. Finally she went to Boston, where she was under Dr. Wesselhœft's care, and the simillimum always produced complete relief from pain.

Dr. Farley—Some years ago I had a similar case. I resorted to Morphia, of which I gave a grain within an hour. It was of no benefit. Then I did what I should have done before, looked for the remedy. Nux vomica$^{15}$ gave relief in five minutes.

Dr. James—Some time ago I was called to see a boy whom I found on his knees in the hallway pressing his hands into his abdomen. He was bent double and could not get up, the pain was so intense. Colocynth.$^{200}$ was given. In five minutes he walked up-stairs and was in bed.

Dr. Powel—Occasionally I have just such attacks. Colocynth., high, always relieves in a few minutes.

Dr. Clark—Some years ago a man came into my office late one night suffering excruciating pain from renal calculi. I found he had had a similar attack the night before. An old-school doctor had given him Morphia and Chloroform. He had been subject to the affection, at intervals, for several years. I gave him Lycopodium$^{cm}$. When he came in the pain was so great that he immediately laid down on the floor. In less than a half-hour after the medicine was given he left the office and walked home, a distance of two miles. For over two years he did not have another attack. Afterward I lost sight of him.

Dr. James—I published a case in the *Organon*, in which a man had excruciating pain in left knee. The pain came suddenly, causing him to fall. I found he wanted to have his leg extended upward in order to get relief. The slightest motion aggravated, yet he was forced to move the leg; must get a new position, yet there was no relief. I gave Pulsatilla, and he was better in a moment.

Dr. Farley—That was a perfect picture of Bryonia.

Dr. James—Dr. Lippe had about that time taught me the difference between Rhus tox. and Bryonia and Puls., Rhus tox. must change to a new position, which relieves for a few moments. Puls., change of position does not relieve. Puls. is better from slow motion, while Rhus is better from rapid motion. Dr. Lippe said Mag. carb. patient is obliged to get up and move about slowly, to obtain relief; he cannot sit still. Mag. carb. has beating, pulsating toothache, pain goes up into cheek, jawbone, and eye, and into the neck; it is insupportable while sitting still.

Dr. Farley—Prosopalgia, with toothache, better from sweat, worse after getting in bed, is chenop. glauc. aphis.

Dr. James—The next case, with regard to relieving pain, was a little girl with a bad form of thrush, which seemed to indicate Merc., which did not relieve. Further study led me to Baptisia, which relieved in one hour.

Dr. Lee—A woman who was in the habit of using Morphia hyperdermically on herself, sent for me to relieve her of painful symptoms. I found her going from one bed to another; she could not keep still. She abused me for not giving her Morphia at once. I gave Ars., which relieved in ten minutes. She acknowledged that the medicine did her much good.

Dr. Farley—Some time ago I went to a dentist who used Arsenic to kill the nerve of a tooth. I became so restless that I could not keep still. I took Ars.$^{40m}$, and was all right in fifteen minutes.

Dr. James—A man took cold from sudden suppression of perspiration. Tonsillitis came on, and his suffering was terrible. His tongue was indented, and saliva ran from his mouth in drops, and he perspired profusely without relief. Merc.$^{cm}$ cured him in two days. An old lady had erysipelas. The inflammation began on the right temple and spread to the left.

The cheek was much swollen and the eye appeared as though pushed back into the head, Lycop. was given. In six hours all pain was gone, and the next day the inflammation disappeared in reverse order to its appearance.

Dr. Farley—In facial erysipelas going from left to right, I gave Rhus tox., and the patient was well in forty-eight hours.

Dr. Lee—A woman had attacks of terrible itching of skin without eruption through three periods of gestation. In the last she complained of the same itching, when Dr. Lippe advised Psorinum. In two days erysipelas appeared. Her face was very much swollen. The child was born on the day after. On the second day the lochia stopped, for which a dose of Bell. was given, and there was no further trouble.

Dr. Farley then presented the following: a baby, æt. eleven months, has had a croupy cough since birth. I was called hastily, Jan. 21st, nine P.M., and found the little one gasping for breath, with harsh, sawing respiration, and clear, ringing, rasping cough. Gave one dose Spongia$^{200}$ dry. In ten minutes the child was quietly sleeping, and the respiration was clearing rapidly. On the morning of Jan. 22d, the father called and said baby seemed all right. At six P. M. I called and detected slight rasping sound and cough, with harsh laryngeal sound. Gave one dose Spongia$^{200}$ dry, and left powder of Hepar$^{30}$, to be given if child grew worse. Jan. 23d, father called and said baby slept nicely until midnight; after that breathing became labored and harsh, and cough ringing and rasping. He had given the Hepar at two A. M.; amelioration followed and continued until eight A. M. From that on the child grew worse. I saw the child at ten A. M., and found the breathing labored and gasping. All the voluntary muscles were being used to aid respiration. Respiration was noisy and rattling, the larynx and chest seeming to be loaded with mucus; cough harsh and rasping, worse from cold water; blood becoming carbonized, as was evidenced by lividity of the face. On examining throat detected small particles of membrane on tonsils, and during cough saw a mouthful of lemon-yellow, muco-purulent matter well up into fauces. The alæ nasi were expanding and contracting markedly at each respiration. I had given a dose of Ant. tart., to be repeated in an hour if not better. Called at twelve M. and found the patient worse, of course. I then went over the case carefully, and got the condition clearly, as above described; I failed to get a clear record at one o'clock. I now gave a dose of Lycop.$^{200}$, and left Sac. lac., to be given every half-hour, and went away, confident of success.

At half-past three o'clock I was hastily called, as the child was said to be worse. Child seemed to be a little worse in every way. The cough was more violent and frequent, respiration more labored and obstructed. I told them she was doing just what I had reason to expect, and that I firmly believed she would get well. I felt sure it was the four to eight P. M. aggravation of Lycop., and determined not to call again until after eight o'clock. Called at half-past eight o'clock, and found the parents happy and the child sleeping peacefully. The breathing was still somewhat labored. Continued Sac. lac.

Jan. 24th, ten A. M., child playing in cradle and seeming perfectly well. Had slept well all night.

---

The 131st meeting of the Lippe Society was held on Tuesday evening, April 9th. Dr. Carleton Smith occupied the chair.

After the minutes of the last meeting were read and approved, the Secretary reported that Dr. Still had sent him a paper on diphtheria to be read at the March meeting; that it had miscarried, and was received only the day before this meeting. The paper was then read. Dr. Smith called special attention to the peculiar symptom of Ignatia in affections of the throat, the suffering worse when not swallowing. It belongs to no other remedy so characteristically, and is always relieved by Ignatia.

Dr. Preston—Some time ago I was called to see a boy aged five years, who had an attack of diphtheria. I had been preceded in the family by allopaths, who had lost two cases. The child was moribund, and his death was expected hourly. I found he was crying all the time except when swallowing. By this peculiar symptom I was led to Ignatia, and he recovered speedily. Ignatia is excellent in croup where the same peculiar symptom is present.

Dr. Powel—I have just successfully treated one of the worst cases of diphtheria I have ever seen. A child, æt. seven years, first taken with scarlatina. The fauces were completely covered with false membrane, also the posterior nares.

As the discharge was excoriating, I first gave Arum-tri., which did no good. Arsenicum$^{40m}$ was then given for the symptoms, which caused improvement. Laryngeal complications then appeared, and for the hoarse, croupy cough, and the characteristic loose, rattling cough I gave Bromine$^{cm}$, a few spoonfuls in water, with excellent results. There was almost total suppression of urine.

Dr. Clark then reported a very interesting case of dysmenorrhœa, calling special attention to the many contradictory symptoms, which seemed characteristic of several remedies.

Dr. Lee said that he knew of a somewhat similar case in old-school hands, in which there was anteflexion of the uterus. After the flexion had been reduced the symptoms were mitigated, but even then oöphorectomy was performed to "cure" the case.

Dr. Preston—I had a case of severe menstrual trouble, which prevented the patient from being out of bed for months. She got the idea that her uterus was displaced, but all that could be done to convince her she was wrong was of no avail. After she had had several attempts made to replace it, without benefit, Pulsatilla made her well.

Dr. Lee said we did wrong not to wait until several menstrual periods had passed before we change the carefully chosen remedy, if that has failed to relieve.

Dr. Farley thought that we should see some sign of improvement in the first period after the appropriate remedy was given.

Dr. Preston—I should wait for at least three periods. If at the end of that time I saw no change for the better I should study the case and choose another remedy.

Geo. H. Clark, *Secretary.*

---

